# Fc*γ*RIIa - dependent platelet activation identified in COVID-19 vaccine-induced immune thrombotic thrombocytopenia-, heparin-induced thrombocytopenia, streptokinase- and anisoylated plasminogen-streptokinase activator complex-induced platelet activation

**DOI:** 10.3389/fcvm.2023.1282637

**Published:** 2023-11-15

**Authors:** Mustapha Abdelouahed, Dana Yateem, Salim Fredericks

**Affiliations:** ^1^ Department of Medical Sciences and Education, Boston University School of Medicine, Boston, MA, United States; ^2^ School of Medicine, The Royal College of Surgeons in Ireland, Medical University of Bahrain, Al Sayh, Muharraq Governorate, Bahrain

**Keywords:** vaccine-induced immune thrombotic thrombocytopenia, COVID-19, platelet activation, Fc*γ*RIIa, heparin-induced thrombocytopenia, vaccination

## Abstract

Coronavirus disease 2019 (COVID-19), which was caused by the coronavirus - severe acute respiratory syndrome coronavirus 2 (SARS-CoV-2), was globally responsible for remarkable morbidity and mortality. Several highly effective vaccines for COVID-19 were developed and disseminated worldwide within an unprecedented timescale. Rare but dangerous clotting and thrombocytopenia events, and subsequent coagulation abnormalities, have been reported after massive vaccination against SARS-CoV-2. Soon after their global rollout, reports of a morbid clinical syndrome following vaccination with adenovirus-DNA-based vaccines appeared. In the spring of 2021, reports of a novel, rare and morbid clinical syndrome, with clinically devastating and fatal complication after vaccination with adenovirus-based coronavirus vaccines (Janssen/Johnson & Johnson and Astra-Zeneca vaccines) led to a brief suspension of their use by several countries. Those complications were associated with unusual cerebral and splanchnic venous thrombosis, and circulating autoantibodies directed against anti-platelet factor 4 (PF4), a protein secreted from platelets, leading to the designation: Vaccine-Induced Immune Thrombotic Thrombocytopenia (VITT). The reported VITT incidence remains very low and does not affect the overall benefit of immunization, however, if left untreated, VITT can be debilitating or even fatal. VITT resembled specific adverse drugs' reactions that also involved the production of autoantibodies and subsequent abnormal platelet activation through platelet Fc*γ*RIIa. These unusual but well-documented drug reactions were heparin-induced thrombocytopenia (HIT), streptokinase- (SK), and anisoylated plasminogen-streptokinase activator complex- (APSAC) associated with platelet-activating antibodies. There was considerable overlapping of clinical features between VITT, COVID-19 and these adverse drugs' reactions. We review the phenomenon of VITT against the backdrop of shared and common mechanisms that underlie HIT-, SK-, and APSAC-platelet Fc*γ*RIIa-dependent platelet activation. An understanding of VITT's pathogenesis may be achieved by comparing and contrasting VITT-, HIT-, SK- and APSAC-induced platelet activation mechanisms, their respective physiopathology and similarities. Discussing these conditions in parallel provides insight into complex immunological disorders and diseases associated with abnormal hemostasis and thrombosis in particular.

## Introduction

Coronavirus disease 2019 (COVID-19), which was caused by the coronavirus - severe acute respiratory syndrome coronavirus 2 (SARS-CoV-2), was globally responsible for remarkable morbidity and mortality. The SARS-CoV-2 virus deploys its “spike protein” in order to gain entry to cells by chemically associating with angiotensin-converting enzyme 2 (ACE2) located on the outer membrane of the cells of the host (1, 2). The trans-membrane protease serine 2 (TMPRSS2), a serine protease, enzymatically cleaves and proceeds to activate the spike protein to fuse the SARS-CoV-2 virus with the cell membrane. This spike protein, a uniquely recognizable virus component, became the target for vaccine manufacturers. Several highly effective vaccines were developed for COVID-19. These vaccines were designed to trigger the immune system, thus resulting in the production of neutralizing antibodies with the ability to identify the SARS-CoV-2 virus. European and US regulatory authorities approved two technological approaches to producing and administering COVID-19 vaccines. These were lipid nanoparticles encapsulating an mRNA payload acting as a template for the SARS-CoV-2 spike protein (BioNTech/Pfizer and Moderna), and replication-attenuated adenovirus vectors carrying a DNA payload encoding the same spike protein (Ad26.COV.2.S or Janssen/Johnson & Johnson and ChAdOx1 nCoV-19 or Astra-Zeneca). All available vaccines, whether they employed mRNA or DNA, led to the host cells' expression of the SARS-CoV-2 spike protein and elicitation of protective immunity (3). The adenovirus-DNA-based vaccines became caught up in a controversy that led to their temporary suspension in several countries. Midway through 2021, reports of a novel, rare and morbid syndrome with devastating and fatal complications after vaccination were published. It was associated with atypically located cerebral and splanchnic venous thrombosis, abnormal platelet activation, and circulating autoantibodies, which attach themselves to the platelet-secreted protein, platelet factor 4 (PF4). This adverse reaction was designated vaccine-induced immune thrombotic thrombocytopenia (VITT) (4–8).

The proposed pathological mechanism of VITT was the production of these antibodies that were directed against the naturally occurring human protein PF4 as a reaction to the vaccine. This led to massive platelet aggregation. It is also conceivable that the thrombocytopenia following vaccination had multiple causes. Other factors may have been necessary for the development of VITT. The proteins ACE2 and TMPRSS2 are abundantly expressed in platelets. These membrane proteins are responsible for spike protein priming (9, 10). Therefore, it was no surprise that platelets would have been involved in the clinical manifestations of COVID-19 disease. However, the function played by platelets in this post-vaccine adverse reaction was more complex than mere virus-protein to host-protein interactions.

Platelets are crucial conduits conjoining the hemostatic system and immune defenses. This includes those immunological mechanisms and processes protecting against viral disease. Platelets also serve as mediators involved in vital elements of inflammatory processes and are instrumental in modulating responses of the immune system to both “self” and “foreign” molecules (11, 12). The cellular phenomenon of platelet activation is known for its crucial contributions to thrombus formation. Hence platelet-activation's role in preventing bleeding and minimizing vascular injury. However, along with this physiological role comes a pathological role, played out in deleterious thrombotic events and associated cardiovascular sequelae. Not dissimilar to innate immune cells, platelets express an array of immune-associated molecules. These include receptor molecules for: IgG's Fc-region, C-type lectin and various complement and chemokine molecules. They also express toll-like receptors (TLRs), and nucleotide-binding and oligomerization domain–like receptors (13, 14). These receptors recognize pathogens such as dengue, human immunodeficiency, and influenza viruses. When in contact with pathogens, platelets mediate immune responses indirectly through the release of cytokines, anti-microbial peptides and directly through interaction with neutrophils, monocytes, and lymphocytes. The net result of which is the amplification of the immune response (15). Furthermore platelets possess a plethora of coagulation factors and inflammatory factors. These are stowed in their α-granules, only to be secreted upon platelet activation to boost further the cascade of events that comprise coagulation (16, 17). Factors V and XIII function as sites of adhesion for the binding of coagulation factors activation via their cell surface exposure to phosphatidylserine (18).

The descriptions of VITT are recent, and research on its pathophysiology is limited. Pathogenicity of VITT may involve both host and vaccine factors (19). However, several grey areas remain regarding knowledge of VITT and its pathophysiology. This poses a stark standout question. How could a strand of DNA coding for a viral protein delivered in an adenovirus vector generate autoantibodies against a human physiologically functional protein such as PF4? The answer may lay in adverse drug reactions involving totally different chemical compounds. The clinical and laboratory details of this adverse reaction to the COVID-19 vaccines revealed intriguing similarities to previously well-documented adverse drug reactions. Platelet activation and the enhancement of platelet activation by autoantibodies through platelet Fc*γ*RIIa is a common feature of VITT with remarkable similarities to heparin-induced thrombocytopenia (HIT), streptokinase (SK), and anisoylated plasminogen-streptokinase activator complex (APSAC) associated platelet-activating antibodies through platelet Fc*γ*RIIa. Accounting for commonalities between the various responses to these medications may expand our knowledge of the pathogenesis of VITT. Comparing and contrasting elements of the VITT response to a vaccine with these other abnormal responses to drugs may allow us to better understand VITT and the mechanisms underpinning adverse reactions to heparin, SK and APSAC. In order to comprehensively assess VITT, in light of the responses to these drugs, it is necessary to review elements of the pathology of COVID-19, the vaccines, platelet function and the role of platelets in the above-mentioned adverse drug reactions.

### COVID-19 and thrombosis

The putative cause of VITT is anti-PF4 antibodies inducing pathological platelet activation through Fc*γ*RIIa, a protein located on the outer membrane of platelets. Circulating immunoglobulin-G antibodies with the ability to induce platelet activation via these Fc*γ*RIIa proteins have also been detected in patients with SARS-CoV-2 virus infection. Therefore, this necessitates a brief exploration of some features of COVID-19 itself in relation to VITT.

Thromboembolic complications are prominent clinical features of COVID-19 (20). Thrombosis in atypical locations, such as cerebral veins, has been reported in patients afflicted with COVID-19. A notable overlap exists in the clinical presentation between VITT and COVID-19, i.e., unusual thrombotic complications have been associated with patients with both conditions. Less severe thrombocytopenia occurs frequently with patients with severe COVID-19 with or without it being leagued with hemostasis activation and systemic inflammation, including fibrinolytic shutdown (21, 22). Substantial proinflammatory aspects, indicate a so-called “cytokine storm”, may often occur with COVID-19 patients. Thus being implicated in the pro-thrombotic syndrome (23). Thrombocytopenia occurred more often in patients with the more severe forms of COVID-19 (24). Twenty per cent of patients with COVID-19 had low platelet counts at the time of hospital admission (25). Among survivors of COVID-19, blood platelet counts were inclined to increase within the second week of hospitalization, whereas, among non-survivors, low blood platelet counts were more inclined to remain low or worsen (26). There is a body of evidence that suggests that COVID-19 predisposes individuals to thromboembolic complications, including arterial and venous thrombotic events, as well as thrombocytopenia. These have all been significantly associated with mortality (27). Reports from several independent research groups described platelets as being hyperreactive during COVID-19 disease, this being coupled with abnormalities of gene expression in platelets. This potentially influences interactions between white blood cells and platelets (28). Quiescent platelets isolated from the blood of patients with COVID-19 had abnormally high expression of P-selectin upon activation. These patients also had raised levels of circulating platelet-monocyte, platelet-neutrophil, and platelet-T-cell aggregates (29–31). Other markers of thrombin generation or activation have been associated with COVID-19. These include thrombin-antithrombin complex as well as prothrombin fragments (32).

The immune system's responses to PF4 involve separate mechanisms from the immune responses to the spike protein of SARS-CoV-2 virus particle. Further, VITT antibodies have been shown not to bind to the spike protein of SARS-CoV-2. This suggests that the response of the immune system to the SARS-CoV-2 spike protein in the vaccine does not induce VITT (33). When discussing VITT, the intended and non-intended immune responses to the vaccine should not be conflated with the immune response to the viral infection of SAR-CoV-2. However, due to the shared and common elements, discussions of the pathogenesis of VITT are here addressed in parallel with issues related to COVID-19 and the responses to specific drugs.

### Vaccine-induced immune thrombotic thrombocytopenia (VITT)

Clinically, VITT is associated with complications related to the hemostatic system (Tables 1, 2). These may be life-threatening thromboses such as deep venous thrombosis, cerebral venous sinus thrombosis, and splanchnic vein thrombosis. These thrombotic events occur paralleled with thrombocytopenia, elevated plasma soluble D-dimer concentrations and raised numbers of activated platelets. Also, there is a detectable amount of anti-PF4-polyanion immunoglobulin antibodies in serum (47, 48). European studies showed an incidence of around 1 per 100,000–250,000 vaccine recipients. More than 90% of these patients were below the age of 60 years and disproportionately female. Fatalities among these VITT patients were reported to be approximately 20%. This relatively low figure may be due to a lag in recognizing and identifying the symptoms and signs by patients and healthcare providers (49).

**Table 1:** Most common complications associated with VITT.

Most common complications associated with VITT	References
Aortic graft thrombosis.	Giacomelli E et al., (34)
Catheter-associated upper-limb DVT.	Bozzani A et al., (35)
Cerebral venous sinus thrombosis (CVST), splanchnic vein thrombosis, pulmonary embolism, deep vein thrombosis, arterial thrombosis, ophthalmic vein thrombosis, intraparenchymal brain hemorrhage, ischemic stroke, and DIC.	Greinacher A et al., (4) Perry RJ et al., (36) de Barry O et al., (37) Hughes C et al., (38) Hemasian H et al., (39) Klein DE et al., (40) Bayas et al., (41)
Other forms of thrombosis, especially venous thromboembolism, abdominal vein clots, and arterial clots.	Rizk JG et al., (42)
CVST, estimated to occur at a rate of 1 per 100 000 vaccine recipients with the Astra-Zeneca vaccine and 1 per 1 000 000 with the Janssen/Johnson & Johnson vaccine. In the US, all patients who received the Janssen/Johnson & Johnson vaccine and developed CVST with VITT were female.	Ropper AH & Klein JP. (43)

**Table 2:** Abnormal laboratory and radiologic abnormalities occurring in VITT.

Abnormal laboratory and radiologic abnormalities occurring in VITT	References
▪ The development of thrombosis at uncommon sites includes CSVT/splanchnic venous thrombosis. It is unclear why this immune-mediated thrombosis manifests mainly in the cerebral vessels and splanchnic circulation. ▪ Presence of VITT autoantibodies, although VITT patients have not received heparin and it is likely that an unidentified polyanion in the adenoviral vaccines or expressed by the infected cells by the vaccine is binding to PF4. In patients with suspected or confirmed VITT, rapid initiation of treatment similar to that of severe HIT is hence recommended. ▪ Mild to severe thrombocytopenia. However, a normal platelet count does not exclude the possibility of VITT in its early stages. ▪ Positive antibodies against PF4 identified by enzyme-linked immunosorbent assay (ELISA) assay. Serologic studies do not show antigen cross-reactivity between anti–SARS-CoV-2 and anti-PF4 antibodies, signifying that anti-PF4 antibodies are unlikely a byproduct of anti-SARS-CoV-2 immunity. Demonstration of circulating anti-PF4 antibodies in conjunction with thrombocytopenia and thrombosis suggests that VITT is a clinical variant of HIT.	Greinacher A et al., (4) Sadoff J et al., (44) Schultz NH et al., (7) See I et al., (8) Oldenburg J et al., (45) Scully M et al., (46)

HIT and the severe form of COVID-19 disease share several common clinical similarities. These two conditions only occur in a small minority of patients infected with SARS-CoV-2 or those patients exposed to heparin. Dissimilar to the related HIT, with an estimated incidence of 1 to 6% of individuals anticoagulated with heparin and a corresponding mortality rate of 25% (50). VITT has been reported as 1:150,000 of Astra-Zeneca recipients and 1:470,000 of Janssen/Johnson & Johnson recipients. These figures carry a mortality rate of 20%–30% for Astra-Zeneca and Janssen/Johnson & Johnson vaccines respectively (51). HIT and VITT are associated with hypercoagulable blood of affected patients and a relatively high occurrence of thrombosis and thrombotic events within atypical blood vessel sites (21). The twain are associated with abnormal full blood cell count values (particularly leukocytes and platelets), coagulation, prothrombin-time, soluble D-dimer plasma concentrations outside the reference intervals, and a potential for disseminated intravascular coagulation. Similar to HIT, VITT has been associated with developing anti-PF4 antibodies. The typical sequela of which may be aggregation of neutrophils and platelets (52).

### Antibodies against PF4 characterize VITT

**Figure 1:**
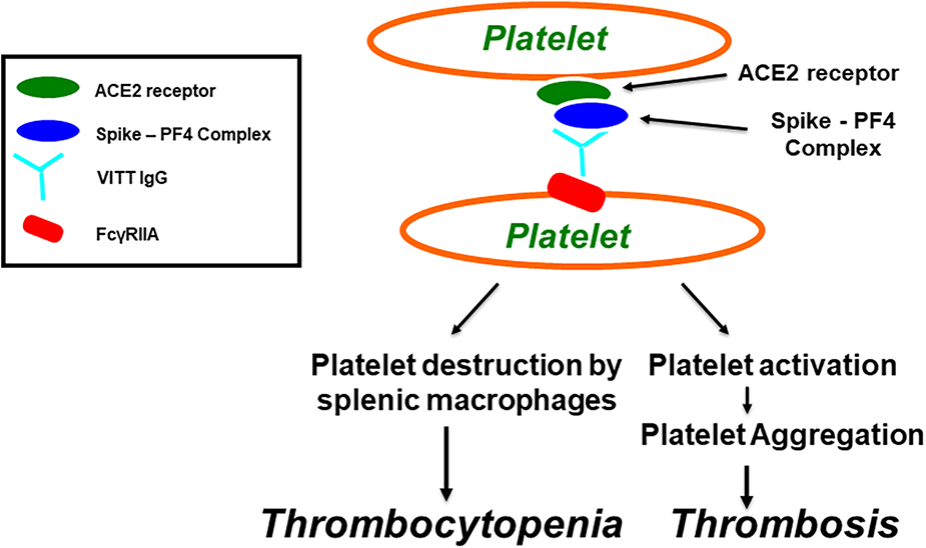
Vaccine-induced immune thrombotic thrombocytopenia (VITT). Antibodies induce platelet aggregation through Fc*γ*RIIa. SARS-CoV-2 virions, or its spike protein, produced after COVID-19 vaccination, bind to platelets via ACE2 receptor, leading to activation of platelets and the secretion of platelet factor 4 (PF4). PF4 then biochemically associates with the spike protein, forming PF4-Spike complexes that stimulate VITT anti-PF4 antibody production. VITT pathological IgG antibodies induce platelet aggregation through platelet Fc*γ*RIIa and thrombocytopenia through platelet destruction by splenic macrophages.

Dissimilar to thrombocytopenia caused by heparin exposure, VITT pathological antibodies are produced independently of exposure to this thrombolytic drug. There are no reports that any of the VITT patients had been previously anticoagulated with heparin prior to the onset of VITT. This suggests that a factor in the vaccine preparation triggered the adverse reaction to the vaccine (45, 53). VITT shares many clinical, laboratory and immunological features with HIT, a pro-thrombotic pathology caused by antibodies that induce platelet-activation by recognizing multimolecular complexes. These structures form between cationic PF4 and anionic heparin (see Figure 1). These features include thrombocytopenia, unusual strokes, anti-PF4 autoantibodies, and platelet activation through platelet Fc*γ*RIIa receptor.

PF4, a 70-amino-acid protein located in α-granules, is secreted from platelets when activated during the aggregation process and promotes further thrombogenesis. PF4 is cationic in its physical nature and acts as a chemokine (54).. Although the primary function of PF4 is to act as a pro-coagulant factor, it is also plays a role in innate immunological and adaptive immunological processes where thrombocytes are activated. This platelet activation is in response to infections. The positively charged PF4 may readily associate with negatively charged bacterial surfaces, thereby promoting, opsonization and contributing defenses against potentially pathological bacterial infection (55). PF4 also forms molecular associations with polyanions, undergoing a structural change in its conformation, which induces anti-PF4/polyanion antibodies (see Figure 1). These help eliminate pathogens (56) and cause HIT in the absence of heparin molecules. This response is termed “spontaneous HIT” (57).

Because VITT patients were not previously exposed to heparin anticoagulation, it is reasonable to suspect a similar mechanism to “spontaneous HIT”. *In vitro* experiments demonstrated that proteolysis of plasma proteins leads to sequential release of endogenous glycosaminoglycans, chondroitin sulfate and heparin (43). The dysregulated proteolysis occurring during COVID-19 or VITT triggers a sustained and excessive release of endogenous chondroitin sulfate and/or heparin that may play a fundamental role in their associated and respective pathophysiology (43). Platelets stimulated by endogenous heparin, either directly or with the intermediation of PF4, release additional heparin may lead to the catastrophic thrombotic thrombocytopenia observed in severe COVID-19 or following vaccination (43).

The pathophysiology of VITT is presumably due to the development of antibodies against PF4 that associates with a highly restricted molecular location on the PF4 protein. This may correspond to the PF4-heparin-binding site. The result of which may be platelet consumption and depletion accompanied by thrombus formation (4, 58). Several published studies have described high levels of pathological VITT anti-PF4 antibodies, present in blood specimens from VITT patients, which induce the activation of platelets through platelet Fc*γ*RIIa receptors in the presence of PF4 without heparin and potentially other cells, through Fc*γ*RIIa receptors (4, 7, 46). The platelet-activating effects of VITT anti-PF4 antibodies can be completely inhibited by anti-Fc*γ*RIIa monoclonal antibodies (59–61). The presence of high concentrations of polyclonal immunoglobulins may interfere with the platelet-activating effects of these autoimmune anti-PF4 antibodies. This may be interpreted as a confirmation of the immune system's role in this hyper-activation of platelets working via Fc*γ*RIIa (61).

There remains the very real possibility that platelet activation contributing to the pathology of VITT may not be solely attributed to anti-PF4 antibodies. Other factors may underpin the processes resulting in these thrombotic events. The progression of HIT may be ascribed to the many pro-thrombotic mechanisms associated with VITT. These include monocyte activation, over production of tissue factor, and pro-coagulant microparticle generation (58). Restricted to eight amino acid sequences that overlap with the heparin-binding site, VITT antibodies associate with molecular regions similar to PF4 as with heparin (58). Anti VITT antibodies may mimic the physiological function of heparin. Thus, assisting in the clustering of the tetramers of the PF4 protein. These are the building blocks of the immune complexes, which bring together Fc*γ*RIIa membrane proteins and induce the activation of platelets (58). It is likely that these VITT pathogenic antibodies associate with and activate other non-platelet cells that also express the mildly ubiquitous Fc*γ*RIIa. Noteworthy are endothelial cells as well as the leucocytes (60). Molecular polymorphisms of Fc*γ*RIIa may influence the risk of VITT and the development of new thrombotic events. These Fc*γ*RIIa polymorphisms impact the affinity of association to the various human IgG subclasses and other molecules involved in Fc*γ*RIIa transmembrane signaling. A similar discussion of Fc*γ*RIIa polymorphisms applies wholly to HIT (62, 63).

It is now well-established that adenovirus engages with and activate platelets (64, 65). Several viruses can directly lead to platelet hyperactivity (66, 67). Further, constituents of Ad-vector COVID-19 vaccines may play the role of “co-factor” binding to PF4, inducing newly formed immunogenic epitopes that potentially induce the production of the pathological anti-PF4 immunoglobulins. The immunoreactivity observed between the vaccine components and platelets or the PF4 protein may be considered a contributor to the genesis and progression of VITT (4, 7). Platelets may be directly be infected by RNA contained within viruses. Therefore RNA from the virus particles may be translated into spike protein via the protein biosynthesis mechanisms possessed by platelets. Thus, initiating an immune response against the body's own platelets (68). Platelets can translate mRNA and synthesize proteins of viral origin, as is seen with dengue, human immunodeficiency, and influenza viruses (69).

### SARS-CoV-2 spike protein chemically associates directly and increases the activation of platelets

The virus SARS-CoV-2 uses receptors in order to infect lung epithelial cells. COVID-19 patients in a critical condition expressing ACE2 as well as TMPRSS2, have hyperactive platelets, mean platelet volume above their reference interval, increased activation of αIIbβ3 and P-selectin expression on thrombocytes (10, 30). MAPK pathway, downstream of ACE2 mediates the potentiating role of SARS-CoV-2 on platelet activation (29). Neutrophils isolated from severe COVID-19 patients demonstrate a clear association with IgG immune complexes. Patients' sera with severe COVID-19 contain high amounts of immune complexes. These complexes potentially activate neutrophils via a mechanism that is partially independent of Fc*γ*RIIa platelets (70).

The Spike protein of the SARS-CoV-2 virus particle binds directly to ACE2 on the surface of platelets, enhancing platelet activation and causing the release of coagulation factors and inflammatory cytokines. In addition, this causes the formation of leukocyte–platelet aggregates and induces thrombus formation (10, 28, 30). Like SARS-CoV-2, through this chemical association with ACE2, the spike protein of SARS-CoV-2 stimulates the secretion of PF4 from platelets and activates cells of the immune system to initiate the inflammatory reaction, thus triggering the production of anti-PF4 antibodies (71).

Structural similarities have been evidenced between platelet factor 4 and SARS-CoV-2′s spike protein (4, 72). The chains are similar in their molecular structural organization, including anti-parallel β-pleated sheets uncased within a pair of α-helices. They both possess common amino acid sequences corresponding to 323–335 residues in RBD and 15–27 amino acidic residues in PF4 (72). Additional molecular similarities between the spike and PF4 have been reported upon, either in the RBD or the other spike protein domains (4). The anti-Spike IgG antibodies do not recognize PF4; the anti-PF4 antibodies demonstrate immunoreactivity with “Spike-RBD” (72). In a similar fashion to heparin-PF4 complexes, since RBD and PF4 can interact with a significant affinity, they form PF4-spike complexes that bind to platelet ACE2 receptors that could have an involvement in the immune production of anti-PF4 antibodies, leading to platelets aggregation through platelet Fc*γ*RIIa (72).

There also remains the possibility that other receptor proteins mediate SARS-CoV-2 spike-platelet interactions. Heparin sulphate, located on the cell surface, is implicated as a binding site for viruses (73). The molecular association of the SARS-CoV-2′s spike protein to cell membrane-bound heparin sulphate was attenuated in the presence of heparin. Thus hindering the passage of viral particles into host cells (74). A substitution mutation within the spike protein at residue 614 (aspartic acid to glycine) has been identified and suggested to be a clear “fitness advantage” in increasing SARS-CoV-2′s transmissibility in The Americas and Europe (75). Platelets interact with the spike protein of SARS-CoV-2 through various C-type lectin receptor proteins and proteoglycans. Variants generated through alternative-splicing of the spike protein exist, as well as for CD147. This being a transmembrane protein highly expressed in blood cells, including platelets. Further, adenovirus vectors also interact with the CD46 receptor.

### VITT: laboratory assessment, prevention, management, and treatment

The reported laboratory aspects associated with VITT point to a consumptive coagulopathy. This involved thrombocytopenia, low plasma fibrinogen concentrations, and elevated plasma concentrations of D-dimer. This was reported with the severest of HIT cases (76). Most reports strongly correlate VITT with the strong presence of anti-platelet factor 4 in serum and platelet activation as assessed by function laboratory assays (4, 7).

Initial testing should be performed for recently vaccinated patients (i.e., between 4 and 30 days post-vaccination) presenting clinically and with new laboratory findings, including a full blood count, soluble D-dimer measures, and plasma fibrinogen measures. Deviations from reference intervals would identify patients with severe manifestations of VITT. New clinical symptoms may include nausea, vomiting, headaches, vision problems, pain in the abdomen and chest, as well as dyspnea (49). If lab findings are within the reference intervals, then there is a low likelihood of VITT. However, these VITT patients should be continually monitored, and laboratory investigations should be repeated if symptoms reoccur or persist. Suppose there is solely a single isolated incident of thrombocytopenia, with plasma soluble D-dimer and plasma fibrinogen within their reference intervals and an absence of thrombosis. In that case, vaccine-induced ITP should be considered a probable diagnosis, particularly when the index patient had received one of the vaccines containing mRNA, supposing there is suggestive evidence of consumptive coagulopathy (e.g., thrombocytopenia and plasma soluble D-dimer) (49). In that case, diagnostic imaging investigations and anti-PF4 IgG immunoassays should be performed to document VITT. If diagnostic imaging investigations reveal thrombosis, hospitalization and the initiation of clinical interventions involving non-heparin anticoagulant drugs are recommended without the transfusion of platelets (49). For complications with hemorrhaging, intravenous immunoglobulin (IVIG) and the steroid prednisone are suggested to resolve the low platelet counts, cryoprecipitate and fresh frozen plasma should be administered to manage coagulopathy (49).

There is sufficient evidence of common clinical and laboratory characteristics observed between VITT and HIT to adopt similar management approaches to the two syndromes (Table 3). Although the pathogenesis of VITT is not yet clear, recently published findings were consistent in describing that the three phenomena of thromboembolic events, low platelet count, and the detection of anti-PF4 antibodies appear as the hallmarks of VITT (9). Society of Thrombosis and Haemostasis Research suggested screening for anti-PF4 antibodies if thromboembolic events or low platelet counts occurs within two weeks post-vaccination (38). Treatment recommendations issued by The American Society of Hematology for VITT are similar to those for severe HIT in patients with low platelet count, thrombosis, and the positive detection PF4-heparin IgG as assessed by immunoassay (38). The regimens for successful management of HIT of autoimmune origin can also be similarly applied to VITT. Interventions may include IVIG and glucocorticoids given in high doses (42).

**Table 3:** VITT: Management and treatment.

Management of VITT	References
Urgent administration of high dose IVIG 1 gram/kg daily for two days. IVIG contains immunoglobulin G of all subclasses derived from pooled human plasma, and has been used in the management of COVID-19. In high doses, IVIG can competitively inhibit the binding of VITT antibodies with the platelet Fc*γ*RIIA, thereby inhibiting platelet activation and aggregation. Case series have documented that IVIG improves platelet count and recovery in VITT by inhibition of serum-induced platelet activation.	Greinacher A et al., (4). Schultz NH et al., (7) Bourguignon A et al., (77) Rizk JG et al., (78)
▪ Evaluation for HIT/VITT should be performed before administering IVIG to prevent false-negative test results due to potential interference of IVIG with ELISA and platelet activation assays. ▪ Given its close resemblance to HIT, the use of heparin may be harmful, and non-heparin anticoagulants should be considered such as: direct thrombin inhibitors: Argatroban or bivalirudin, direct oral anti-Xa inhibitors without heparin bridge: Edoxaban, apixaban, dabigatran or rivaroxaban, low molecular weight heparinoid devoid of heparin: Danaparoid, and selective factor Xa inhibitor: Fondaparinux. Restricting catheter-directed fibrinolysis to experienced centers and patients whose condition deteriorates despite intensive coagulation. Systematic reviews have shown that fibrinolytic treatment is associated with intracranial and extracranial major bleeding complications. Thus, fibrinolytic therapy should only be considered in patients with extensive CVST whose condition worsens despite anticoagulation.	Saposnik G et al., (79) Canhão P et al., (80)
Other treatment recommendations for VITT ▪ Hematology specialist consultation must be sought as early as possible. ▪ IVIG and non-heparin-based anticoagulation should be initiated if there is a high suspicion for the diagnosis of VITT and PF4-ELISA results are pending. ▪ Platelet transfusions should be avoided and should be considered only if clinically indicated and at the advice of a hematologist. ▪ Fibrinogen must be corrected with fibrinogen concentrate or cryoprecipitate to maintain a fibrinogen level of >1.5 g/L. ▪ All forms of heparin that include heparin-based flushes must be avoided until VITT is ruled out. ▪ Patients should be transferred to a tertiary care center once the diagnosis of VITT is confirmed. ▪ Both the BSH and ASH recommend continuing systemic anticoagulation for a minimum of three months in patients with documented thrombosis. ▪ Steroids can inhibit the synthesis of new autoantibodies, thereby interrupting platelet activation and thrombosis. More recently, systemic steroids have been used in combination with IVIG for the management of VITT. Of the 5 individuals with VITT in the study by Schultz et al., 4 were treated with concomitant methylprednisolone (1 mg/kg/d) and IVIG. Three patients had documented CVST and 2 of these patients died. George et al., reported successful treatment for a patient with VITT and CVST using steroids, IVIG, and bivalirudin. Antiplatelet therapies may be warranted while therapeutic intervention targeting the cytokine storm in severe COVID-19 is gaining increasing attention, the use of antiplatelet therapy also warrants further study in treating COVID-19 patients to improve patient outcome. Aspirin administration has been associated with a reduced risk of mechanical ventilation, intensive care unit admission, and in-hospital mortality in 412 hospitalized COVID-19 patients.	Schultz NH et al., (7) Manne BK et al., (30) George G et al., (81) Mehta P et al., (82) Chow JH et al., (83)

### Platelet Fc*γ*RIIa activation associated with HIT

**Figure 2:**
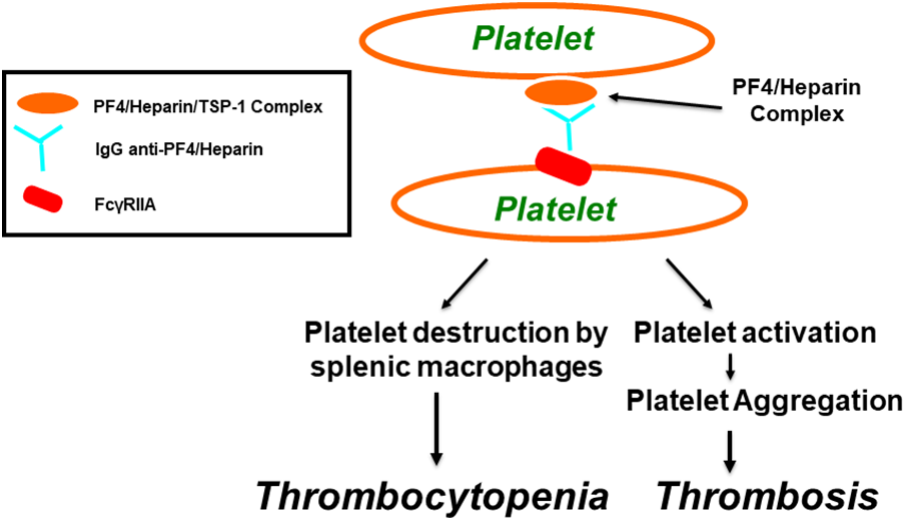
Pathogenesis of heparin-induced thrombocytopenia (HIT) as well as thrombosis through FcγRIIa. Within α-granules of platelets, PF4 is associated with a proteoglycan carrier that contains chondroitin-4-sulfate. Platelet factor 4 biochemically associates with physiologic concentrations of heparin, resulting in the molecular formation of the PF4/heparin combined complexes that leads to generation of anti-PF4/heparin antibodies that lead to the generation of anti-PF4/heparin antibodies. Multimolecular complexes of PF4-heparin and antibodies lead to cross-linking of FcγRIIA, inducing platelet aggregation and thrombocytopenia, through platelet destruction by splenic macrophages.

Both VITT and HIT disorders are associated with thrombocytopenia, thrombosis, the presence of autoantibodies to PF4, and platelet activation through platelet Fc*γ*RIIa. The key adverse drug reaction to the anticoagulant heparin is HIT. It is a pharmacologically induced immunoglobulin-mediated thrombocytopenic disorder associated with both arterial and venous thrombosis resulting from the generation of platelet thrombi (84, 85). Approximately 5% of patients who receive heparin anticoagulation therapy develop HIT. Out of those patients, about 5%–80% develop thrombosis, which can involve arterial vessels, venous vessels, or both vessel types (86–88). HIT is characterized by the presence of platelet-activating antibodies that recognize multimolecular complexes between the cationic PF4 and the anionic heparin, leading to platelet activation through Fc*γ*RIIa receptors, resulting in thrombosis, endothelial cell injury, and the release of procoagulant-rich microparticles (see Figure 2) (89–93). The removal of platelets via phagocytosis by splenic macrophages, in a Fc*γ*RIIa-dependent mechanism, or platelet consumption caused by thrombi formation, can explain thrombocytopenia (4, 94).

Thrombotic risk in HIT is strongly correlated with high levels of circulating anti-PF4 antibodies, as detected by immunoassays and functional platelet activation assays (95–97). Platelet activation induced by antibody-PF4/heparin complexes is accompanied by other cascading reactions that result in thrombotic complications (98). HIT immune complexes also activate other cells, such as monocytes (99), neutrophils (100), and endothelium (89, 101). HIT antibodies cross-link Fc*γ*RIIa on platelets, monocytes, and neutrophils, initiating pro-coagulant cellular responses that generate a profound hypercoagulable state (102). Although HIT is considered a platelet activation disorder, neutrophilia is common, particularly in thrombosis patients (103).

### Platelet Fc*γ*RIIa activation associated with SK and APSAC

Platelet activation and the enhancement of platelet activation by autoantibodies binding to platelet membrane Fc*γ*RIIa is a common feature of VITT (47), HIT (4), SK, and APSAC (59, 104–107). Both SK and APSAC thrombolytic drugs act predominantly by bringing about the biomolecular transformation of inactive plasminogen to physiologically and biochemically active plasmin. This, in turn, breaks down the physicochemical structures of both fibrin molecules and fibrinogen molecules, thus achieving thrombolysis.

Streptokinase, an exogenous plasminogen activator derived from bacteria, has the latent potential to trigger an innate immune response, subsequently producing antibodies against SK. These anti-SK antibodies inhibit the breakdown of fibrin whilst simultaneously inducing platelet activation. These antibodies are highly prevalent among particular populations, such as individuals exposed to β-hemolytic streptococci infection or those with previous pharmacological exposure to SK (108). Following SK exposure, there is a progressive increase in detectable plasma levels of anti-SK antibodies, reaching a maximum at around two weeks post-SK thrombolytic therapy (109).

**Figure 3:**
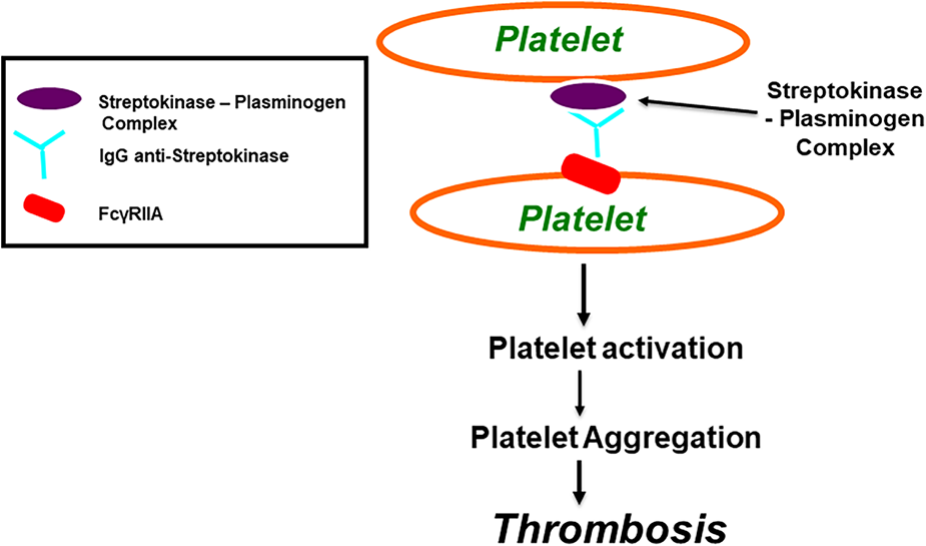
Streptokinase (SK) and Anisoylated Plasminogen-Streptokinase Activator Complex (APSAC) induced platelet aggregation through FcγRIIa. Both Streptokinase and APSAC modify in vitro platelet aggregation by two mechanisms; reduced aggregation due to fibrinogenolysis, and enhanced aggregation via an immunological reaction. The reduced aggregation by SK (or APSAC) is mediated by plasmin generation and the fibrinogen degradation product, fragment E. As shown in this figure, SK (or APSAC) may also trigger platelet aggregation by a mechanism involving specific IgG anti-SK. Both SK- (or APSAC) induced platelet aggregation and SK- (or APSAC) enhanced ADP-induced platelet aggregation require the interaction of the Fc domain of the anti-SK antibodies with the platelet FcγRIIA.

Both SK and APSAC modify the *in vitro* platelet aggregation predominantly via two main mechanisms. These being fibrinogen breakdown (107) and immune responses (see Figure 3) (59, 104–107). When SK or APSAC are added to normal subjects' platelet-rich plasma (PRP), there is an inhibition of “platelet aggregation” by way of response to platelet-activating pharmacological agonists [e.g., adenosine diphosphate (ADP) and collagen] (104, 106, 110). The SK or APSAC inhibition effect is mediated by plasmin generation, as it is attenuated in the presence of aprotinin (110). Platelet aggregation inhibition by SK is caused by the fibrinogen fragment E (110). Using PRP from healthy blood donors, the prevalence of SK- (or APSAC) reducing the aggregation of platelets by ADP was found to be 47% (104).

SK or APSAC also potentially induce platelet aggregation via specific circulating anti-SK IgG antibodies (104, 107) (see Figure 3). A previous infection with streptococcus or a history of treatment with SK (or APSAC) may influence the presence or absence of a patient's circulating anti-SK antibodies. Awareness of the broader deleterious effects of SK and APSAC may prove paramount when selecting from the array of available thrombolytic therapies so as to minimize the risk of excessive blood loss or thrombosis (104).

Using the PRP collected from healthy donors, we previously reported 5.8% and 53% prevalences for thrombolytic-associated “platelet aggregation” for streptokinase and APSAC, respectively (104). This thrombolytic drug-associated “platelet aggregation” reaction was accompanied by the release of a relatively large concentration of 4.5 µmol/L ATP. However, without streptokinase-induced “platelet aggregation”, activation of platelets occurred to some extent with SK-induced platelet shape change only with the release of a mere 0.05 µmol/L ATP (104). SK and APSAC-inducing platelet aggregation or enhancing of platelet aggregation are unaffected by the presence of aprotinin, indicating that the observed effect is specifically associated with SK and works independently of the generation of plasmin, a product from its plasminogen precursor (104). In PRP, SK- or APSAC-induced platelet aggregation showed no discernable correlation with the amount of healthy subject plasma anti-SK antibodies (104). A higher titre of anti-streptokinase antibodies was bereft of any such association with platelet aggregation in response to SK or APSAC exposure, using *in vitro* experimental settings, suggesting that anti-SK antibodies- activating platelets constitute a clearly defined sub-group of whole anti-SK antibodies in plasma (104).

Platelets perform a pivotal part in stimulating the response of the immune system in bringing about the said thrombolytic drug-induced “platelet aggregation” in the SK- (or APSAC) “enhanced ADP-induced platelet aggregation” as demonstrated by our in vitro experiments (59, 107). This was evidenced by mixing IgG anti-SK, isolated from patients recently diagnosed with an acute myocardial infarction who received SK for thrombolysis, and washed platelets from apparently healthy volunteer donors (59, 107). The combination of platelets from healthy subjects, “responders” to platelet-activating monoclonal antibody PL2-49, with SK (or APSAC) and purified IgG-induced platelets aggregation whilst simultaneously enhancing the SK platelet aggregation response to ADP. This response is mediated via platelet Fc*γ*RIIa and accompanied by the generation of inositol triphosphate, the subsequent cellular influx of Ca^2+^, and TXA2 release (59, 107). Further, compounds known to inhibit the catalytic activities of protein tyrosine kinase (PTK), as well as the enzyme protein kinase-C (PKC) and the biological catalyst phospholipase-C (PLC), have been employed to investigate “platelet aggregation” as induced by “anti-SK antibodies” and additional APSAC in isolated washed platelets. These compounds being: erbstatin - a PTK inhibitor; GF 109203X - a PCK inhibitor; and neomycin – an inhibitor of PLC. The presence of these enzyme inhibitors prevented the aggregation of platelets induced by “anti-SK antibodies” in addition to APSAC, suggesting that PTK, PCK and PCL pathways are fundamental to activating platelets via the immune system (59). The TXA2 receptor antagonist – SQ 29548 and the cyclooxygenase inhibitor – aspirin significantly attenuated the activation of platelets in this *in vitro* experimental setting (59). The release of ADP also contributed considerably to the “platelet aggregation” response, as confirmed by employing apyrase, a compound known to attenuate the immunoglobulin-G anti-streptokinase with the addition of APSAC-induced aggregation of platelets (59).

Two distinct pathways and their dynamic interactions enable “anti-SK antibodies” to elicit streptokinase-induced “platelet aggregation” and “streptokinase-enhanced platelet aggregation”. These pathways are: (i) the activation of platelets by the immune system via membrane-bound Fc*γ*RIIa, and (ii) the involvement of a demarcated subgroup of thrombocytes eliciting antibodies directed against SK (59, 104–107).

Fc*γ*RIIa structural polymorphisms correlate with platelet's functional response following stimulation and activation by antibodies of mouse origin or aggregated in response to antibodies of human origin (111). Several monoclonal antibodies induce the activation of platelets via interactions between their Fc-domains and membrane Fc*γ*RIIa. Platelets from apparently healthy donors demonstrate a vast variation in response to activating monoclonal antibodies (112–116). Although the mechanisms involved in these response disparities have not been definitively elucidated, the majority of reports addressing the platelet Fc*γ*RIIa functional polymorphism were performed in PRP (112, 113, 116, 117).

In our previous PRP studies, all platelets from subjects with SK- (or APSAC) elicited aggregation of platelets and SK- (or APSAC) enhanced “platelet aggregation” induced were assigned as “responders” in response to the monoclonal activating antibody PL-249 (88, 89). However, all subjects with only streptokinase (or APSAC) enhanced platelet aggregation were considered “intermediate-responders” and as an alternative group, “non-responders” in the biological reaction of these cohorts to the PL-249 (88, 89). However, in washed platelets, SK- (or APSAC) induced platelet aggregation and SK- (or APSAC-) “enhanced ADP-induced platelet aggregation”, observed using platelets from “responders”, “intermediate-responders” or “non-responders” to activating monoclonal antibody PL-249, indicating that the functional platelet Fc*γ*RIIa polymorphism is observed only when platelets are activated in PRP and not observed when using washed isolated platelets (104, 105). Because the platelet Fc*γ*RIIa functional polymorphism was observed only in PRP and not in washed platelets, we suggested that other important plasma factor(s) and mechanisms are involved in platelet Fc*γ*RIIa functional polymorphism (105).

The risk of developing thrombosis in VITT may depend on platelet Fc*γ*RIIa polymorphism; this has been reported to be a risk factor for HIT (62, 63). Further, *in vitro* studies and investigations may be needed to determine the role of platelet Fc*γ*RIIa polymorphism in VITT by comparing the platelet aggregation elicited *in vitro* by purified VITT IgG anti-PF4 in PRP vs. washed platelets, including platelets from different apparently healthy subjects defined as “responders”, “intermediate-responders” or “non-responders” to mouse activating platelet antibodies; such as the PL-249 monoclonal antibody.

### VITT incidence rates: COVID-19 Oxford/AstraZeneca and Johnson & Johnson/Janssen vaccines and their relation to VITT

VITT is very rare, however, it can be life threatening, especially if the diagnosis and treatment are delayed. It remains highly relevant in countries that afforded and can only afford adenoviral vector-based vaccines for COVID-19 vaccination campaigns. The vaccination campaign, combined with an educational program informing the public and especially physicians about VITT symptoms and appropriate treatment, allowed a reduction in mortality from 50% to 5% - 6% in Australia (118). In Table 4, we focus on the COVID-19 Oxford/AstraZeneca and Johnson & Johnson/Janssen vaccines, used in the Western world with most reported VITT cases. The VITT incidence caused by COVID-19 Oxford/AstraZeneca and Johnson & Johnson/Janssen vaccines varies by the reporting country.

**Table 4:** Incidence of VITT in relation to COVID-19 Oxford/AstraZeneca and Johnson & Johnson/Janssen vaccines (119).

Manufacturer	Oxford/AstraZeneca	Johnson & Johnson/Janssen
Vaccine efficacy	63%	67%
VITT Incidence, as of Q1 2022	1/64,000–1/125,000	1/310,000–1/200,000
VITT mortality rate, as of Q1 2022	18%	15%
Total number of vaccines administered in the USA or EU (in millions)	67	37
Suspension	▪ In late February 2021, reports of thrombosis with thrombocytopenia occurring after vaccination led to the temporary suspension of its use in multiple European countries. ▪ By 10 March 2021, 30 cases of thromboembolic events in response to five million vaccinations were reported by the European Medicines Agency.	▪ The U.S. Food and Drug Administration (FDA) and Centers for Disease Control and Prevention (CDC) suggested pausing the administration of Johnson & Johnson/Janssen in April 2021 due to six reports of TTS after 6.8 million doses were administered. ▪ In December 2021, the CDC recommended against the use of the vaccine. ▪ In May 2022, the FDA officially limited the authorized use of Johnson & Johnson/Janssen to adults for whom other authorized COVID-19 vaccines were not accessible or clinically appropriate, or for adults who declined other authorized COVID-19 vaccine options.

### Worldwide population and demographics reaction to COVID-19 vaccines

Vaccination is critical for controlling the COVID-19 pandemic. As of 30 June 2022, 66.4% of the world's population had received at least one dose of a COVID-19 vaccine, however only 17.4% of people in low-income countries had received a first dose, underlying unequal access, availability and delivery (120–122). Despite the reductions in COVID-19 disease severity, hospitalizations and deaths, since the introduction of multiple safe and effective COVID-19 vaccines, vaccine hesitancy and refusal remains substantial, caused in part by misinformation, lower education, mistrust in science and governments, and elusive broad public support (123). The study of COVID-19 vaccine hesitancy among 23,000 respondents in 23 countries during 2022, representing almost 60% of the world's population, showed willingness to accept vaccination at 79.1%, however one out of eight (12.1%) vaccinated respondents are hesitant about booster doses (123). Twice-yearly COVID-19 booster vaccinations are recommended in some countries based on eligibility and availability. The same factors that influence hesitancy to accept an initial COVID-19 dose also drive booster hesitancy (124). COVID-19 vaccine hesitancy persists for vaccine boosters, which may present a serious challenge to anticipated routine COVID-19 immunization programs. Worldwide, policymakers and public health officials must address COVID-19 vaccine hesitancy and resistance as a component of their overall prevention and vaccination strategy.

## Discussion & conclusion

Vaccines are vital and invaluable to control pandemics and to build up herd immunity. They have considerably reduced the incidence of childhood illnesses, notably measles, mumps, and rubella, and have facilitated the eradication of many infectious diseases, such as poliomyelitis, diphtheria, and smallpox (9). From the onset of COVID-19 pandemic to November 2021, more than 771 million cases and 6,960,7835 million deaths were documented worldwide. Vaccination for prevention against COVID-19 is crucial in containing the spread of SARS-CoV-2 and controlling this pandemic. For the Covid-19 pandemic, the use of diverse technologies allowed the development and production of highly effective vaccines, all produced with unprecedented timelines. The two most common COVID-19 vaccine platforms currently in use are the messenger RNA (mRNA) and adenovirus vector vaccines. The development of antibodies directed against the SARS-CoV-2 spike protein is the strategy chosen by most vaccine developers.

The overwhelming inflammatory response in patients with SARS-CoV-2 infection can lead to VITT associated with a hypercoagulable state, thrombosis, large vessel thrombosis, and, ultimately, death. VITT's overwhelming inflammatory bodily reaction to COVID-19 vaccination was observed in a small minority of vaccine recipients. The risk of VITT associated with thrombosis and mortality should be considered considerably subordinate to the relative risk of loss of life and morbidity attributable to the SARS-CoV-2 infection. VITT recognition raised concerns regarding the safety of COVID-19 vaccines and led to the reconsideration of vaccination strategies in many countries. Prior to vaccination, individuals should be educated about the clinical symptoms associated with VITT syndrome and should be advised to seek urgent medical treatment. VITT appears to be similar to HIT-, SK-, and APSC - induced platelet activation through platelet Fc*γ*RIIa, all showed strong, dose-dependent respective antibodies-mediated platelet activation, which is completely inhibited by IV.3, an Fc*γ*RIIa-blocking monoclonal antibody. In the event of major thrombotic events 4–30 days after COVID-19 vaccination, VITT should be considered; its treatment consists of therapeutic anticoagulation with nonheparin anticoagulants, and the infusion of high-dose IVIG to neutralize the anti - PF4 antibody-Fc*γ*RIIa binding. Routine platelet transfusions, aspirin, and warfarin should be avoided because of the possibility of worsening thrombosis and magnifying bleeding risk. There are no well-known risk factors for VITT with CVST post vaccination.

VITT was observed in both males and females across a wide age group, thus, there may not be a gender or age restriction in regard to suspected VITT. Therapeutic anticoagulation with nonheparin anticoagulants is the primary treatment for VITT with or without CVST. Given the concern of VITT, healthcare providers should be familiar with the VITT clinical presentations, pathophysiology, diagnostic criteria, and management consideration. Furthermore, they should maintain a high index of suspicion in patients presenting with symptoms on laboratory abnormalities suggestive of VITT as early recognition and management of this syndrome can prevent catastrophic complications. With increased VITT awareness and recognition by providers and the public alike, future cases should help refine VITT clinical understanding and improve its clinical outcomes. The better understanding of the mechanisms behind the VITT thrombotic complications is needed to aid in better disease management and improve the prognosis. Vigilance should be maintained regarding large-scale injections of vaccines, which contain adenoviruses and genetic material from the SARS-CoV-2 spike protein.

In immune complex disorders, platelets interact with IgG-containing immune complex through platelet Fc*γ*RIIa. The similarities of the mechanisms of VITT-, HIT-, SK, and APSAC-induced platelet activation could be relevant for a better understanding of immune diseases associated with abnormal hemostasis and thrombosis in general, and to VITT in particular. The exact VITT pathogenic mechanism is yet to be fully elucidated, further, *in vitro* as well as *ex vivo* experiments, investigations and specific lines of inquiry are warranted to reveal the true relationship that lies between COVID-19 vaccinations with VITT, and VITT possible risk factors in order to implement risk minimization strategies.
