# Corrigendum: FcγRIIa—dependent platelet activation identified in COVID-19 vaccine-induced immune thrombotic thrombocytopenia-, heparininduced thrombocytopenia, streptokinase- and anisoylated plasminogen-streptokinase activator complex-induced platelet activation

**DOI:** 10.3389/fcvm.2023.1342177

**Published:** 2023-12-06

**Authors:** Mustapha Abdelouahed, Dana Yateem, Salim Fredericks

**Affiliations:** ^1^ Department of Medical Sciences and Education, Boston University School of Medicine, Boston, MA, United States; ^2^ School of Medicine, The Royal College of Surgeons in Ireland, Medical University of Bahrain, Al Sayh, Muharraq Governorate, Bahrain

**Keywords:** platelet activation, COVID-19, vaccine induced immune thrombotic thrombocytopenia, vaccination, FcγRIIa

A Corrigendum on FcγRIIa—dependent platelet activation identified in COVID-19 vaccine-induced immune thrombotic thrombocytopenia-, heparin-induced thrombocytopenia, streptokinase- and anisoylated plasminogenstreptokinase activator complex-induced platelet activation by Abdelouahed, M., Yateem, D., and Fredericks, S. (2023). *Front. Cardiovasc. Med*. 10:1282637. doi: 10.3389/fcvm.2023.1282637

In the published article, there was an error in Figure 1 as published. The incorrect image for Figure 1 was used. The corrected figure appears below.

**Figure 1:**
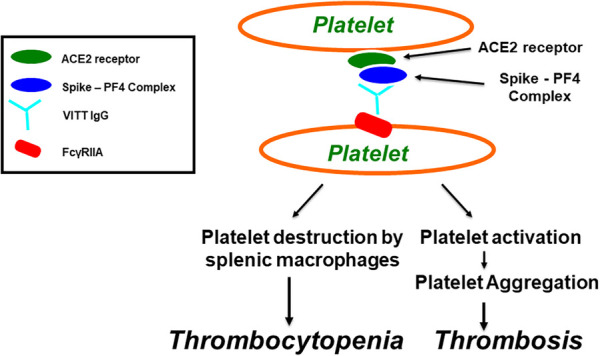
Vaccine-induced immune thrombotic thrombocytopenia (VITT). Antibodies induce platelet aggregation through Fc*γ*RIIa. SARS-CoV-2 virions, or its spike protein, produced after COVID-19 vaccination, bind to platelets via ACE2 receptor, leading to activation of platelets and the secretion of platelet factor 4 (PF4). PF4 then biochemically associates with the spike protein, forming PF4-Spike complexes that stimulate VITT anti-PF4 antibody production. VITT pathological IgG antibodies induce platelet aggregation through platelet Fc*γ*RIIa and thrombocytopenia through platelet destruction by splenic macrophages.

In the published article, there was an error in Figure 3 as published. The incorrect image for Figure 3 was used. The corrected figure appears below.

**Figure 3:**
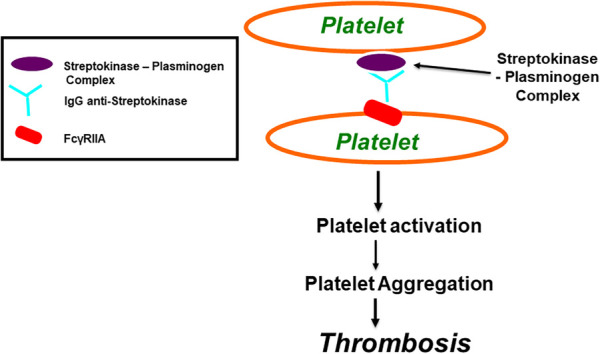
Streptokinase (SK) and Anisoylated Plasminogen-Streptokinase Activator Complex (APSAC) induced platelet aggregation through FcγRIIa. Both Streptokinase and APSAC modify in vitro platelet aggregation by two mechanisms; reduced aggregation due to fibrinogenolysis, and enhanced aggregation via an immunological reaction. The reduced aggregation by SK (or APSAC) is mediated by plasmin generation and the fibrinogen degradation product, fragment E. As shown in this figure, SK (or APSAC) may also trigger platelet aggregation by a mechanism involving specific IgG anti-SK. Both SK- (or APSAC) induced platelet aggregation and SK- (or APSAC) enhanced ADP-induced platelet aggregation require the interaction of the Fc domain of the anti-SK antibodies with the platelet FcγRIIA.

The authors apologize for these errors and state that this does not change the scientific conclusions of the article in any way. The original article has been updated.

